# Liposomes are Poorly Absorbed *via* Lung Lymph After Inhaled Administration in Sheep

**DOI:** 10.3389/fphar.2022.880448

**Published:** 2022-06-02

**Authors:** Jibriil P Ibrahim, Shadabul Haque, Robert J Bischof, Andrew K Whittaker, Michael R Whittaker, Lisa M Kaminskas

**Affiliations:** ^1^ School of Biomedical Sciences, University of Queensland, St Lucia, QLD, Australia; ^2^ Monash Institute of Pharmaceutical Sciences, Monash University, Parkville, VIC, Australia; ^3^ School of Science, Psychology and Sport, Federation University, Berwick, VIC, Australia; ^4^ Australian Institute for Bioengineering and Nanotechnology, School of Chemistry and Molecular Biosciences, The University of Queensland, St Lucia, QLD, Australia

**Keywords:** lung lymph, pharmacokinetics, sheep, inhalation, mediastinal lymph, Nanoparticles, liposomes

## Abstract

Enhancing the delivery of therapeutic agents to the lung lymph, including drugs, transfection agents, vaccine antigens and vectors, has the potential to significantly improve the treatment and prevention of a range of lung-related illnesses. One way in which lymphatic delivery can be optimized is *via* the use of nanomaterial-based carriers, such as liposomes. After inhaled delivery however, there is conflicting information in the literature regarding whether nanomaterials can sufficiently access the lung lymphatics to have a therapeutic benefit, in large part due to a lack of reliable quantitative pharmacokinetic data. The aim of this work was to quantitatively evaluate the pulmonary lymphatic pharmacokinetics of a model nanomaterial-based drug delivery system (HSPC liposomes) in caudal mediastinal lymph duct cannulated sheep after nebulized administration to the lungs. Liposomes were labelled with ^3^H-phosphatidylcholine to facilitate evaluation of pharmacokinetics and biodistribution in biological samples. While nanomaterials administered to the lungs may access the lymphatics via direct absorption from the airways or after initial uptake by alveolar macrophages, only 0.3 and 0.001% of the ^3^H-lipid dose was recovered in lung lymph fluid and lymph cell pellets (containing immune cells) respectively over 5 days. This suggests limited lymphatic access of liposomes, despite apparent pulmonary bioavailability of the ^3^H-lipid being approximately 17%, likely a result of absorption of liberated ^3^H-lipid after breakdown of the liposome in the presence of lung surfactant. Similarly, biodistribution of ^3^H in the mediastinal lymph node was insignificant after 5 days. These data suggest that liposomes, that are normally absorbed *via* the lymphatics after interstitial administration, do not access the lung lymphatics after inhaled administration. Alternate approaches to maximize the lung lymphatic delivery of drugs and other therapeutics need to be identified.

## Introduction

The lung lymphatic system is responsible for regulating and optimizing lung fluid levels, (Weber et al., 2018), modulating local immune defenses (Bromley et al., 2005) and regulating the progression of some pulmonary diseases (Trevaskis et al., 2015). It is therefore now widely accepted as an important target for drug delivery applications, particularly for cancer chemotherapeutics and immunomodulators. To this end, nanomaterial-based drug delivery systems, such as liposomes, nanoparticles and dendrimers, have become important facilitators for improved drug access to the lymph after interstitial and, in some cases, intravenous administration (Kaminskas et al., 2009; Azzi et al., 2016; Ke et al., 2019; Marasini and Kaminskas, 2019; Siafaka et al., 2021; Zheng et al., 2021). After inhaled administration however, our understanding of lung lymphatic absorption and trafficking of nanomaterials is limited and research is sparse. Further, there is conflicting information in the literature regarding whether nanomaterials traffic towards the lung lymphatics after inhaled administration. This in part comes from a lack of quantitative lymphatic pharmacokinetic data and use of small animal models that have different pulmonary pharmacokinetics compared to humans and larger animals, particularly for macromolecules and nanomaterials (Choi et al., 2010; Ryan et al., 2016).

Using rodent models, previous papers have proposed that nanoparticles enter the lung lymphatics via non-cellular lymphatic draining or antigen-presenting cell (APC) mediated trafficking (Choi et al., 2010; Trevaskis et al., 2015). Optimal nanomaterial properties for lung lymphatic access after pulmonary administration, for example, have been reported to be smaller than 30 nm and exhibit a non-cationic charge (Choi et al., 2010). Lung lymphatic access of a 200 nm solid lipid nanoparticle however, has also been reported in rats after pulmonary administration (Videira et al., 2002; Choi et al., 2010). Liposomes (typically around 100–200 nm diameter) modified to mimic the bacterial wall have also been proposed to be efficiently absorbed *via* the lung lymph after pulmonary administration in pigs and allow radiometric visualization of the lung lymphatic network (Botelho et al., 2011; Liu et al., 2020). However, 10 nm dendrimers have been shown to exhibit poor absorption via the lung lymph after inhaled administration in sheep (Videira et al., 2012). Specifically, while a 22 kDa PEGylated dendrimer showed 16 and 9% pulmonary bioavailability in rats and sheep respectively after nebulized administration to the lungs, and 30% absorption via the lymph after interstitial administration, less than 0.5% of a nebulized dose was quantified in the lung lymph of sheep over 7 days (Ryan et al., 2016). This contradicted prior studies in rats and pigs that suggested good lymphatic absorption of inhaled nanomaterials. The latter study in sheep, however, quantified lung lymphatic pharmacokinetics by continuously collecting lung-derived lymph from the efferent caudal mediastinal lymph duct (CMLD) that collects the majority of lung-derived lymph before it enters the thoracic lymph duct (Chanana and Joel, 1985). The former studies in rats and pigs however, used non-quantitative imaging-based approaches.

While most nanomaterial structures are far from clinical translation as inhalable nanomedicines, there are currently over a dozen inhalable liposome-based formulations in the clinic and clinical trials for a range of diseases (Haque et al., 2016). Further, the inhaled delivery of liposome and non-liposome-based vaccines has the significant potential to enhance local mucosal immunity in the lungs compared to conventional interstitial injection. As an example, the PEGylated liposome-based Moderna and Pfizer Covid-19 vaccines would be expected to provide enhanced immunity and protection against Covid-19 after inhaled administration and may also minimize the risk of myocarditis compared to conventional intramuscular injection (Marasini et al., 2017; Abhyankar et al., 2021; Fanciullino et al., 2021; Li et al., 2021). It is therefore important to better understand the absorption and clearance mechanics of liposomes and other nanomaterials in the lungs to establish whether it is feasible to enhance the lung lymphatic delivery of drugs and other therapeutics *via* this route (Haque et al., 2016).

The objective of this work was therefore to quantify the lung lymphatic pharmacokinetics of ^3^H-labelled non-PEGylated conventional soy phosphatidylcholine (HSPC) liposomes using the established CMLD cannulated sheep model. While PEGylation can improve liposome stability in the lungs, lung lymphatic exposure was previously reported after inhaled administration of non-PEGylated nanoradioliposome (Botelho et al., 2011). Further, non-PEGylated liposomes dominate the inhalable nanomedicines pipeline and are more likely to be internalized by alveolar macrophages that can assist in trafficking the liposomes to the lymph, making them an appropriate investigational tool for this study.

## Methods

### Materials

Hydrogenated Soy Phosphatidylcholine (HSPC, Coatsome NC-21E) was purchased from Yushi-Sheihin (Singapore). ^3^H-dipalmitoyl phosphatidylcholine (ART0532) was from American Radiolabeled Chemicals (MO, United States). Cholesterol, Evans blue dye and urea assay kits (MAK006) were from Sigma-Aldrich (NSW, Australia). Bupivicaine, diazepam and heparin were each purchased from Clifford Hallam Healthcare (Vic, Australia). Saline (0.9% NaCl) was obtained from Baxter (NSW, Australia). Procaine penicillin, cephazolin and Lethabarb^TM^ were from Virbac (NSW, Australia). Transdermal Fentanyl patches were obtained from Janssen Pharmaceuticals (Beerse, Belgium). Isoflurane was purchased from Delvet (NSW, Australia). Thiopentone was from Troy Laboratories (NSW, Australia). Polyvinyl catheters (1.5 mm × 2.7 mm) and endotracheal tubes (Portex, 7–8mm i.d.) were purchased from Smiths Medical (Australia), while silastic tubing (0.63 mm × 1.19 mm) was purchased from Dow Corning (MI, United States). Soluene, Ultima Gold^TM^ and scintillation vials were from PerkinElmer (MA, United States). All other reagents were AR grade and were used without further purification.

### Preparation and Characterization of Liposomes

Liposomes were prepared *via* lipid film hydration as previously described (Haque et al., 2018). Briefly, HSPC (30 mg), cholesterol (8 mg) and ^3^H-dipalmitoyl phosphatidylcholine (250 µCi) were dissolved in chloroform (7 ml) and sonicated for 60 s using a VibraCell sonicator at 50% power (Fischer Scientific, Illkirich, France). This solution was then evaporated to dryness under reduced pressure using a rotary evaporator (Buchi Laabortechnik, Switzerland) at 60°C to form a dry, thin lipid film. Dried lipid films were then rehydrated in phosphate buffered saline (PBS pH 7.4) and agitated for 2 h at 40°C and re-sonicated for 60 s. After rehydration, the lipid solution was extruded using 7 passes through a mini-extruder set (Avanti Polar Lipids, Al, United States) using polycarbonate filters of pore diameter 200 nm, and 100 nm sequentially to form unilamellar liposomes. Unincorporated ^3^H radiolabel (as degraded phosphatidylcholine) was removed from the final liposome formulation by centrifuging liposomes for 5 min at 10,000 x g through a 3 kDa MWCO spin filter (Sigma Aldrich, Australia). Liposomes were then characterized for size, polydispersity and charge on a Malvern Zetasizer (Nano ZS, Malvern Instruments, Worcestershire, United Kingdom). Final characteristics were (as mean ± sd, *n* = 3) size: 161 ± 3 nm, PDI: 0.13 ± 0.03, charge: −2.47 ± 0.4 mV, specific radioactivity 6.5 uCi/mg lipid. Transmission electron cryomicroscopy (cryo-TEM) was also performed on non-radiolabeled liposomes and the image is shown in the supplementary information. Cryo-TEM confirmed the size, spherical and predominantly unilamellar nature of the liposomes.

To examine the stability of the liposomes during nebulization, non-radiolabeled liposomes were nebulized over 20 min in the PARI vibrating mesh nebulizer (sealed on either end of the chamber with parafilm to prevent venting) and analyzed via DLS. Nebulization led to a small increase in liposome size (30 nm) and PDI (0.16), but charge was not significantly affected (liposomes remained relatively uncharged). The results are shown in the supplementary information.

### Animals

Female merino (*ovis aries*) sheep (approximately 28–35 kg, 1–2 years of age, *n* = 10) were sourced through the Monash Animal Research Platform, Monash University and acclimatized for 1 week in communal indoor pens. Sheep were housed under ambient conditions (20–22°C) on a 12 h light dark cycle with food and water provided *ad libitum.* Sheep were fasted for 12 h prior to surgery. Sheep remained in metabolism cages during post-surgical recovery and for the duration of the study. All experimental procedures were approved by the Monash University Animal Ethics Committee and were conducted in accordance with the Australian Code of Practice for the Care and Use of Animals for Scientific Purposes.

### Surgical Cannulation of Sheep

The preparation, and surgical cannulation, of sheep under isoflurane anesthesia were conducted as described previously with some modification to antibiotics and analgesics used (details of pre- and post-surgical infection control and pain relief are reported in the supplementary information) (Enkhbaatar et al., 2003; Ryan et al., 2016; Kaminskas et al., 2020). Briefly, the right jugular vein was initially cannulated in all sheep to allow for saline (0.9% NaCl) administration during and after surgery (during the recovery phase—24–48 h post surgery), intravenous (IV) dosing and serial blood sample collection over the experimental phase (0–5 days post liposome administration). Sheep were then cannulated *via* the efferent CMLD *via* thoracotomy to continuously collect lung-derived lymph for pharmacokinetic analysis. All sheep underwent thoracotomy, but only sheep whose CMLD was well resolved underwent surgical cannulation of the CMLD (6 sheep). In all other sheep where cannulation of the CMLD was not possible, the rib cage was closed without further manipulation and these sheep were used as surgical “sham” controls that were subsequently used to evaluate intravenous pharmacokinetics for calculation of pulmonary bioavailability. Of the six sheep where the CMLD cannulation was attempted, four sheep were successfully cannulated.

The CMLD was cannulated with silastic tubing via a thoracotomy as previously described (Ryan et al., 2016). The cannula was exteriorized through a small chest incision to allow continuous collection of lymph into heparinized vessels. Sheep were then returned to individual metabolic cages to recover while on IV saline. Sheep were dosed with liposome 2–3 days after surgery.

### Liposome Administration and Sample Collection

Prior to liposome administration, samples of “blank” blood (5 ml), CMLD lymph, feces and urine were collected. Blood samples were collected *via* the jugular vein cannula into citrate-EDTA tubes, while lymph drained continuously into heparinized tubes.

CMLD-cannulated sheep (to be dosed *via* the lungs) were then transferred to a body sheath to restrain animals during dosing and collection of bronchoalveolar lavage fluid (BALF). Immediately before pulmonary dosing, a sample of pre-dose BALF was collected *via* a catheter placed in a lung lobe in CMLD-cannulated sheep using a fiber-optic endoscope to guide catheter placement as previously described (Ryan et al., 2016; Kaminskas et al., 2020). BALF samples were centrifuged at 4°C for 10 min (3,500 x g) to collect cell-free BALF and the cell pellet. Cell pellets were then mixed with 1 ml MQ water and frozen for storage and to disrupt the cell membranes.

Sheep were then administered 1 mg/kg liposome (as lipid, approximately 200 µCi ^3^H per sheep) in sterile saline *via* IV administration over 30 s (5 ml dose in non-CMLD cannulated sheep followed by 20 ml saline to flush through any remaining dose), or *via* nebulization (in 5 ml final volume in CMLD cannulated sheep). Nebulized administration was achieved by inserting a cuffed endotracheal tube into the trachea, *via* the nasal passage, and attaching the end of the tube to a respirator (Harvard Apparatus, MA, United States) to control breathing (20 breaths per minute, 1:2 IDE ratio). Liposome was then aerosolized and delivered to the lungs using a PARI eFlow® Inline vibrating mesh nebulizer (PARI, Gräfeling, Germany) until no dose remained (approximately 20 min). Immediately after the completion of aerosol dosing, post-dose BALF samples were collected from an alternate lung lobe to the pre-dose sample and processed as described above. Immediate post dose blood samples (5 ml) were also collected from all sheep (IV and pulmonary dosed), with further serial blood samples collected over the next 120 h. Once dosing was complete, all tubing used for nebulized administration, the nebulizing bulb and exhaled air filter were collected and rinsed with water to calculate the ^3^H dose not delivered and retained in the lungs. Pulmonary pharmacokinetics were then determined based on the actual dose delivered to the lungs, calculated as nominal ^3^H dose minus ^3^H dose recovered in nebulization equipment after completion of dosing as described previously (Ryan et al., 2016).

Plasma was isolated from blood via centrifugation at 3,500 x g for 10 min. Lymph was collected over post dose intervals 0–5 min, 5–30 min, 30–60 min, 1–2 h, 2–4 h, 4–6 h, 6–8 h, 8–12 h, 12–24 h, and daily thereafter. Lymph samples were centrifuged for 10 min at 3,500 x g to separate lymph fluid from the cell pellet (containing alveolar macrophages and other immune cells) that were subsequently processed as described above. Further BALF samples were also collected from pulmonary dosed sheep 24, 72 and 120 h after dosing to describe the rate of ^3^H-liposome clearance from the lungs and processed as described above. Feces and urine were collected continuously as described previously (Ryan et al., 2016).

After the last samples had been collected, sheep were euthanized *via* a bolus injection of Lethabarb (20 ml) *via* the jugular vein cannula. The lungs, liver, kidney, spleen and caudal mediastinal lymph node (CMLN) were then collected, weighed and tissue samples collected and stored at −20°C for further analysis.

### Quantification of ^3^H-Lipid in Plasma, Organ, Urine and Feces Samples

The ^3^H content of biological samples were evaluated as previously described with some modification and details of the analysis described in the supplementary information (Thomson, 1999; Boyd et al., 2006; Ryan et al., 2016). After quantification of ^3^H content in biological samples, the mass of ^3^H-lipid from liposomes in each sample was determined using the specific activity of the liposomes.

Since BALF samples were diluted in saline, the actual concentration of ^3^H in BALF was determined using the urea correction method as described previously (Kaminskas et al., 2020). BALF data are represented as the % change in BALF concentration compared to the sample collected immediately after the completion of dosing.

### Pharmacokinetic Analysis and Statistics

Concentrations of ^3^H liposome have been expressed as ng/ml and were calculated based on the specific activity of the liposomes. This approach, for the purpose of pharmacokinetic calculations, assumes that ^3^H is entirely associated with intact liposomes, which is not always the case. Pharmacokinetic data therefore has to be evaluated with this caveat. This is an inherent issue in quantifying biodegradable nanomaterials with no intrinsic chromophore.

Non-compartmental pharmacokinetic parameters (including elimination rate constant, k; half life; area under the curve, AUC; apparent volume of distribution, Vz; clearance, Cl; maximum plasma concentration and time to maximum plasma concentration, Cmax and Tmax respectively) were calculated using PKSolver (Zhang et al., 2010). The fraction of the dose recovered in plasma was calculated by dividing AUC^0-∞^_pulm_ by AUC^0-∞^_IV_. The cumulative proportion of the pulmonary dose recovered in lymph over time was calculated by determining the mass of ^3^H recovered in each sample and converting this to a % of the delivered dose. The % recovered at each time was added to the % recovered at all prior times to give the cumulative curve.

Statistical analysis was performed using GraphPad Prism, with statistical significance determined at a level of *p* < 0.05. Concentrations of plasma ^3^H between pulmonary and IV administration, and between plasma and lymph concentrations were compared *via* two-way ANOVA followed by Sidaks multiple comparison test. Differences organ biodistribution and excretion of ^3^H in urine and feces were compared between IV and pulmonary delivery via unpaired Students T-tests.

## Results

### Plasma Pharmacokinetics

**FIGURE 1:**
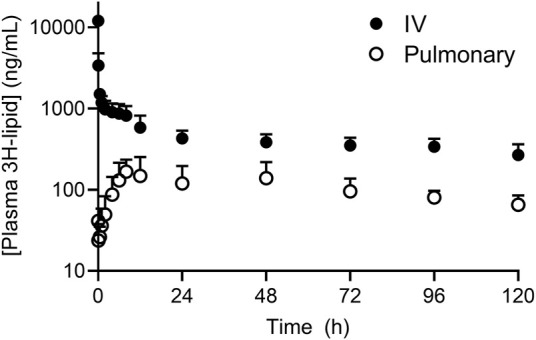
Plasma concentration-time profile of ^3^H-lipid after administration of radiolabeled (^3^H-phosphatidylcholine) liposomes to sheep via intravenous (*black circles*) or pulmonary (*open circles*) administration. Plasma concentrations were normalized to a dose of 1 mg/kg. Data represent mean ± SD (*n* = 4).

The plasma concentration-time profile of ^3^H-lipid (after dosing liposome) after IV and pulmonary administration in sheep are presented in Figure 1 and pharmacokinetic parameters are reported in Table 1. After IV dosing, plasma concentrations declined rapidly over the first hour to give a large apparent volume of distribution (Table 1), but showed slower elimination thereafter. The elimination half-life was calculated to be approximately 5 days (Table 1). Only approximately 20% of the dose was recovered in excreta, suggesting prolonged retention of the ^3^H-lipid in the body.

**TABLE 1:** Plasma pharmacokinetic parameters of ^3^H-liposomes after IV or pulmonary administration to sheep. Data are normalized to a dose of 1 mg/kg in all sheep and are presented as mean ± s.d. (*n* = 4). *Represents *p* <0.05 cf. IV *via* unpaired students T-test.

		IV		Pulmonary	
Parameter	Unit	Mean	SD	Mean	SD
k	h^−1^	0.006*	0.003	0.011*	0.003
t_1/2_	h	126*	43	63*	17
T_max_	h	NA	NA	23	18
C_max_	µg/ml	NA	NA	0.2	0.08
AUC ^0-t^	(µg/ml)h	51*	11	13*	6.0
AUC ^0-inf_obs^	(µg/ml)h	104*	42	18*	7.8
Vz_obs	µg/(µg/L)	56	6.7	NA	NA
Cl_obs	µg/(µg/L)/h	0.35	0.17	NA	NA
dose in urine	%	7.2	3.1	3.2	1.5
dose in feces	%	12.1	3.1	8.9	2.7
F_plasma_	-	NA	NA	0.17	0.13

After pulmonary administration, plasma concentrations steadily increased with a Cmax of 200 ± 80 ng/ml at 23 h (Table 1). Thereafter, plasma concentrations decreased with an elimination half-life of 63 ± 17 h, approximately 2-fold more rapid than the elimination half-life after IV administration. The apparent bioavailability of the pulmonary ^3^H dose was 17 ± 13%. Approximately 3 ± 1.5% and 9 ± 2.7% of the ^3^H dose was respectively recovered in the urine and feces over 5 days after dosing. Cumulative excretion data over 5 days after pulmonary dosing are provided in the supplementary information and show appearance of radiolabel predominantly over the first 2 days after doing.

### Lung Retention Time

**FIGURE 2:**
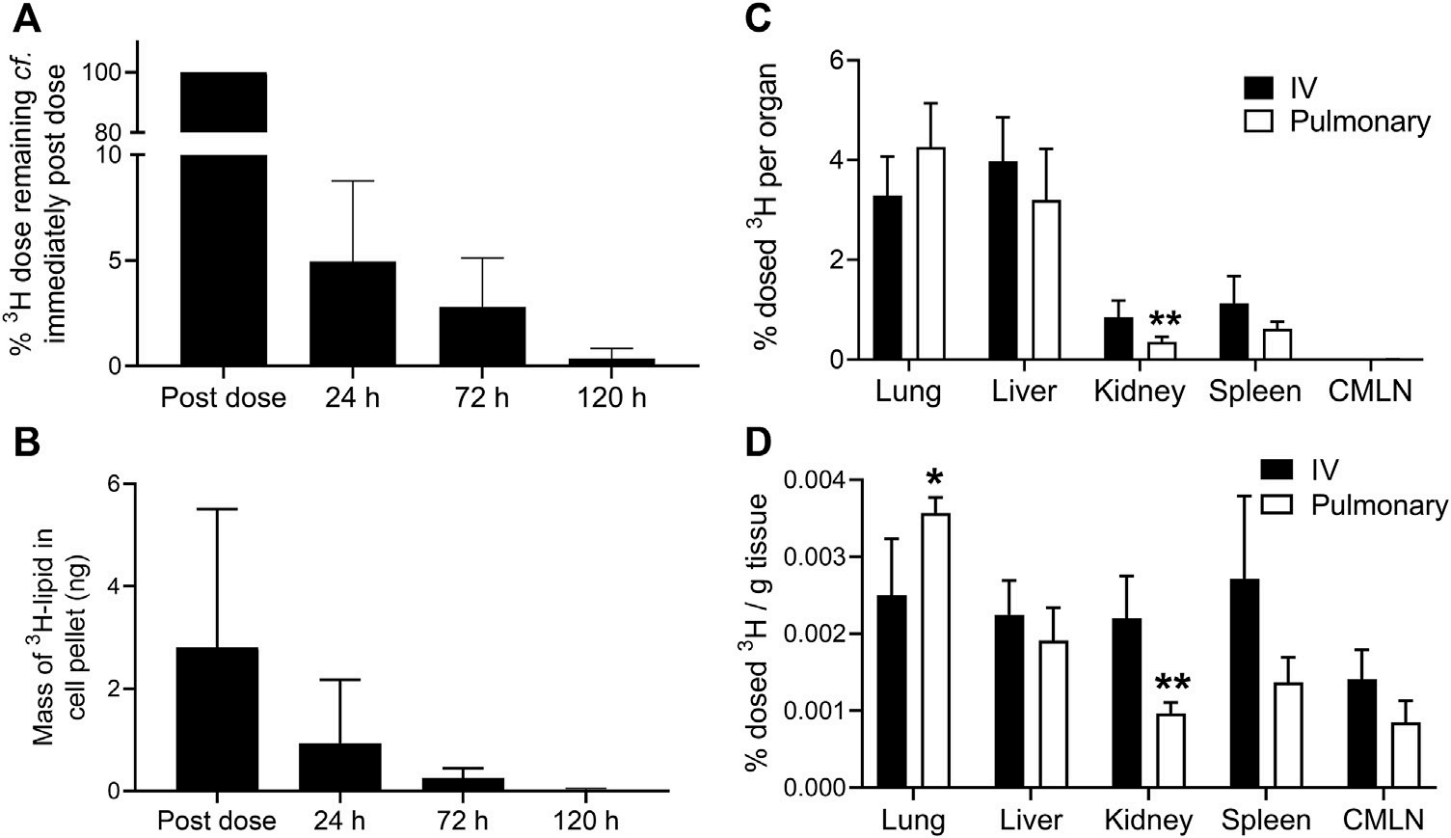
Biodistribution of ^3^H-lipid after IV or pulmonary administration of ^3^H-liposomes to sheep. **(A)** % Change in ^3^H-lipid concentration of cell-free BALF in sheep over time compared to immediately after the completion of pulmonary dosing. **(B)** Mass of ^3^H-lipid recovered in BALF cell pellets over time after pulmonary delivery. **(C)** Biodistribution of dosed ^3^H-lipid in whole organs and tissues 5 days after via intravenous or pulmonary dosing. **(D)** Mass normalized biodistribution of dosed ^3^H-lipid (% dose per gram of tissue) 5 days after IV or pulmonary dosing. Data represent mean ± SD (*n* = 4). * P<0.05, ** P<0.01 compared to IV dosing *via* two-way ANOVA with Sidaks Multiple Comparison test.

The rate of elimination of the ^3^H-lipid dose from BALF is shown in Figures 2A and is represented as % dose remaining in BALF compared to immediately post-dose. These data showed that 95 (± 4)% of the delivered ^3^H dose was eliminated from the BALF within the first 24 h. Thereafter, BALF concentrations steadily declined such that less than 1% of the dose initially deposited in the BALF remained after 5 days. The ^3^H content of the BALF cell pellet, which is comprised largely of alveolar macrophages, was separately analyzed and data are shown in Figure 2B. These data showed that the ^3^H content of the BALF cell pellet only declined by approximately 65% compared to immediate post dose, likely reflecting the slow uptake of liposomes by alveolar macrophages. Beyond 24 h though, a similar pattern of ^3^H elimination from the BALF cell pellet was apparent compared to cell-free BALF.

### Biodistribution

After 5 days, major organs and the CMLN were collected for biodistribution analysis (Figures 2C,D). Despite the low apparent bioavailability of pulmonary dosed ^3^H, no significant differences in absolute ^3^H-lipid biodistribution (*p* >0.05) were observed between IV and pulmonary dosed sheep, with the exception of kidneys (*p* <0.05). The ^3^H concentration of lungs was significantly higher in pulmonary dosed sheep, but only on a per gram basis. Less than 0.01% of the dose was recovered in the CMLN after 5 days and did not differ between pulmonary and IV dosed sheep.

The organ:plasma ratios (calculated from ng lipid/g of sample and reported in the supporting information) show higher concentrations of lipid in tissues at 5 days compared to plasma. Organ:plasma ratios were significantly higher in the pulmonary dosed group compared to the IV group for lungs (12.7 ± 2.2 vs. 3.4 ± 2.2 respectively) and liver (6.6 + 0.4 vs. 3.0 ± 1.5 respectively), but not in other organs.

### Lung Lymphatic Pharmacokinetics

**FIGURE 3:**
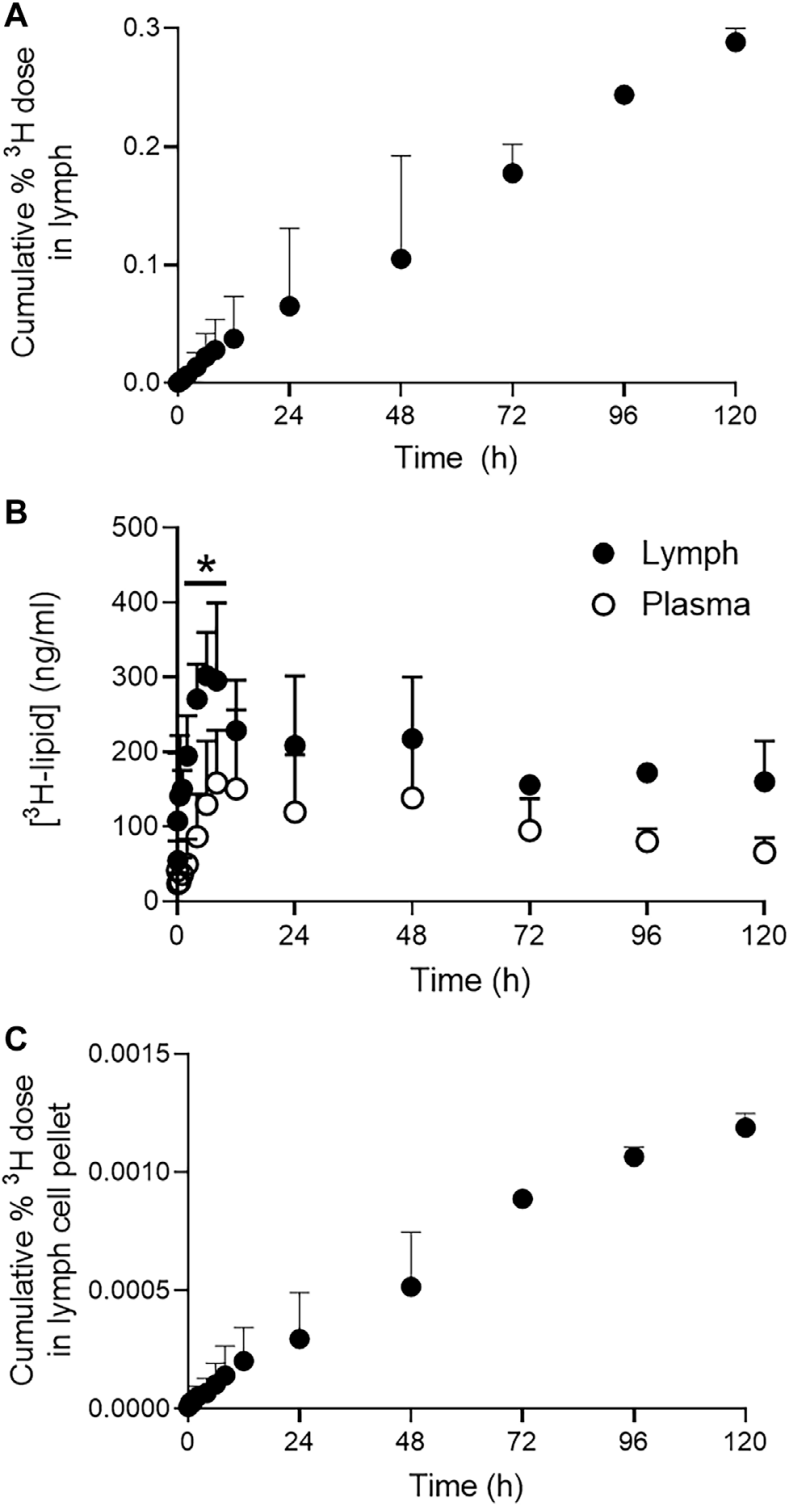
Pulmonary lymphatic pharmacokinetics of ^3^H-lipid in CMLD-cannulated sheep after nebulized administration of ^3^H-liposomes. **(A)** Cumulative recovery of dosed ^3^H-lipid in CMLD lymph after pulmonary administration. **(B)** Comparative concentrations of ^3^H-lipid in collected lymph (*black circles*) and plasma (*open circles*). Concentrations in plasma and lymph were normalized to a dose of 1 mg/kg. **(C)** Cumulative recovery of dosed ^3^H-lipid in lymph cell pellets after pulmonary administration. Data represents mean ± SD (*n* = 4). * P<0.05 between samples at each time point *via* two-way ANOVA with Sidaks Multiple Comparison test.

The cumulative recovery of the ^3^H dose in cell-free CMLD lymph after pulmonary administration is shown in Figure 3A. Despite plasma and lymph concentrations reaching a peak around 6–23 h after pulmonary administration (Figure 3B), the cumulative recovery of ^3^H-lipid in lung lymph increased in a linear manner over 5 days and showed no evidence of plateauing by the last sample collected. Peak lymph concentrations were approximately 300 ng/ml and declined to approximately 180 ng/ml after 5 days. While concentrations of ^3^H in CMLD lymph after pulmonary dosing were approximately 2-fold higher compared to plasma obtained from the jugular vein, only 0.3 ± 0.02% of the delivered dose was recovered in CMLD lymph over 5 days.

Since nanomaterials can access the lung lymph either passively *via* the interstitium, following initial passage through the pulmonary endothelium, or after uptake by local phagocytic cells (in particular, alveolar macrophages), the ^3^H content of lymph cell pellets were separately quantified. The cumulative recovery of the ^3^H dose in the lymph cell pellets reflected the linear profile apparent for the lymph fluid (Figure 3C). However, only 0.002% of the ^3^H dose was collectively recovered in the lymph cell pellets after 5 days compared to 0.3% via the lymph fluid.

## Discussion

The pulmonary lymphatic system is an important drug and vaccine target due to its involvement in disease progression and immune regulation (Pereira et al., 2015). There is therefore growing interest in developing approaches to enhance drug and vaccine access to the lung lymphatic system. In general, the pulmonary lymphatic system has a hybrid role, acting to regulate fluid levels within the lung to facilitate efficient gas exchange while also protecting the lungs *via* immune mediated pathways (Bromley et al., 2005; Weber et al., 2018). This is facilitated in part by the presence of lymphatic vessels primarily in the interalveolar septa in the deep lung and the large airways (Partanen et al., 2000; Weber et al., 2018). Together, this allows for a bi-directional flow of lymph; from the lung perimeter to the pleura and from the lung interior to the hilum, allowing the deeper lung lobes to clear faster than the upper lobes despite reduced lymphatic reach (Schraufnagel, 2010). However, the blood vascular supply of the lungs is significantly denser, particularly around the alveoli. This, together with the very tight cellular junctions in the lungs (Weber et al., 2018), particularly in the alveolar region, can limit the capacity of macromolecules and nanomaterials to access the lung lymphatics after pulmonary administration.

We have previously shown poor lymphatic absorption of a 10 nm PEGylated dendrimer following pulmonary administration, contradicting previously held assumptions that the lung lymphatic network mediates nanoparticle absorption (Ryan et al., 2016). In the present study, we again found that less than 0.5% of a pulmonary dose of HSPC liposome was absorbed into the lung lymphatic network. Given that pulmonary bioavailability was calculated to be approximately 17%, this suggests that less than 2% of the absorbed liposome dose was absorbed via the lymph. While the concentration of ^3^H-lipid in lung lymph was approximately 2-fold higher than in plasma, this does not contravene the suggestion of limited lymphatic availability. This is because the ^3^H content of lung lymph is derived primarily of lipid that has gained access to the lymph *via* absorption directly from the lungs, thereby representing “local” lymphatic concentrations. Plasma sampled from the jugular vein however, is systemic, not specifically lung derived, and ^3^H-lipid absorbed from the lungs *via* the blood is therefore subject to a more significant dilution effect compared to in CMLD lymph.

Despite this, given that ^3^H-lipid was still steadily being absorbed into lung lymph after 5 days, while less than 5% of the dose remained in the lungs at this point, the data suggest there is likely to be a degree of lymphatic recirculation occurring in the lungs. Specifically, since the plasma and lymph concentration profiles are very similar, it appears that a reasonable proportion of ^3^H-lipid absorbed *via* the blood is distributed back into the interstitium of the lungs and thorax, and made available for lymphatic reabsorption as suggested previously for PEGylated dendrimers (Kaminskas et al., 2009). Alternatively, ^3^H-lipid may be redistributed into lymph nodes via high endothelial venules (HEVs) that innervate lymph nodes and play a key role in lymphocyte transport (Ager, 2017). Nanoparticles have been shown to extravasate out of blood vessels in a size dependent manner in a microfluidic model and may enter the lymphatic circulation through HEVs, and this mechanism is now being explored as a possible method for optimizing intravenous drug delivery to lymph nodes (Kaminskas et al., 2009; Vu et al., 2019). Mouse model studies using nanoparticles engineered to target HEVs have already shown improved accumulation in lymph nodes after intravenous delivery (Azzi et al., 2016; Bahmani et al., 2018).

While lymphatic redistribution has typically been described for nanomaterials, this can also occur with lipids that bind to hydrophobic pockets in carrier proteins or chylomicrons, and as such, lipids have been developed as lymphatic targeting tools and drug delivery systems (Trevaskis et al., 2015; Han et al., 2016). To this end, the primary ^3^H species likely absorbed from the lungs into plasma and lymph was ^3^H-phosphatidylcholine or a metabolite, and not intact liposome. While liposomes have some capability to cross membranes, they are more likely to be adsorbed by cell membranes or, in the case of the lungs, be degraded by surfactants or oxidation/hydrolysis processes (Glukhova, 2020). This can be established by analyzing the ^3^H content of biological samples *via* size exclusion chromatography (SEC), as has been done previously in rats (Haque et al., 2018). However, due to the low level of radioactivity in plasma and lymph samples in this study, SEC analysis of samples was not possible. After IV and pulmonary administration of this liposome composition in rats, though, we formerly showed that the predominant ^3^H species in blood after IV administration was intact liposome. By 24 h however, and in all plasma samples examined after pulmonary administration, smaller species (including protein-associated ^3^H) dominated the SEC profiles, including a species that co-eluted with ^3^H-phosphatidylcholine (Haque et al., 2018). This likely also occurred here in sheep. Interestingly though, the elimination half-life of ^3^H in sheep after pulmonary administration was considerably shorter compared to after IV administration (2 vs. 5 days respectively). Further, while pulmonary bioavailability, was calculated to be only 17%, excreta and organ biodistribution data suggested higher bioavailability (> 50%, consistent with that found after pulmonary administration of this liposome composition in rats). Collectively, this points towards the possibility that the ^3^H species absorbed from the lungs and found in plasma after IV administration differed, resulting in the lower observed bioavailability. This may be due to differences in liposome and lipid erosion/biodegradation pathways between the lungs and blood.

The active uptake of non-PEGylated liposomes by alveolar macrophages and lymphatic immune cells may possibly explain the limited lymphatic exposure of liposomes after pulmonary administration (Haque et al., 2018). While the proportion of the ^3^H-lipid dose found in CMLD lymph fluid over 5 days was limited, as mentioned above, liposomes can also presumably traffic into the lung lymph after initial uptake by phagocytic cells, such as APCs or alveolar macrophages. However, only 0.002% of the pulmonary dose was quantified in total collected lymph cell pellets over 5 days. Further, only 7% of the ^3^H label quantified in total BALF (fluid and cell pellet) was identified in the cell pellets (data not shown) and 1.5% of the dose was found in the CMLD after 5 days. This collectively suggested that non-PEGylated, and non-targeted liposomes are not significantly transported into lung lymph by APCs or macrophages over 5 days, despite the fact they are avidly internalized by these cells (Haque et al., 2018; Haque et al., 2019). This was interesting given that alveolar macrophages have previously been reported to transport nanoparticles to lung draining lymph nodes (Kirby et al., 2009). Attempts to expand on this discovery, however, have been disappointing, with one mouse study showing only dendritic cells are capable of mediating transport of nanoparticles into draining lung lymph nodes (Hardy et al., 2013). It is worth noting, however, that the cellular composition of lymph fluid was not measured in this study.

In conclusion, this study quantitatively demonstrates that inhaled HSPC liposomes are poorly absorbed *via* the lung lymphatics. While previous studies have shown the potential for lung lymphatic access after pulmonary administration in rodents, or after inhaled administration of nanoradioliposomes that reflect the bacterial cell wall in pigs, we found less than 0.5% of the pulmonary dose was absorbed into the lung lymphatic network. Further, lymphatic exposure of ^3^H was likely a result of a combination of lymphatic absorption of products of liposome degradation and lipid metabolism, and lymphatic redistribution from the systemic circulation. This highlights the need to look for alternative approaches to enhance the delivery of drugs and other therapeutic agents towards the lung lymph after inhaled administration. While it is feasible to target nanomaterials to the lung lymphatics by relying upon lymphatic redistribution after IV administration, this approach results in higher systemic exposure and related off-target effects.

## Data Availability

The raw data supporting the conclusions of this article will be made available by the authors, without undue reservation.
