# Supplementary material for: Liposomes are Poorly Absorbed via Lung Lymph After Inhaled Administration in Sheep
Source: Front Pharmacol. 2022 Jun 2;13:880448. doi: 10.3389/fphar.2022.880448 (PMC9201389; doi:10.3389/fphar.2022.880448)
Supplement: Supplementary file 2 [file DataSheet1.docx]

Liposomes are poorly absorbed via lung lymph after inhaled administration in sheep

# Characterization of liposome morphology and stability to nebulization


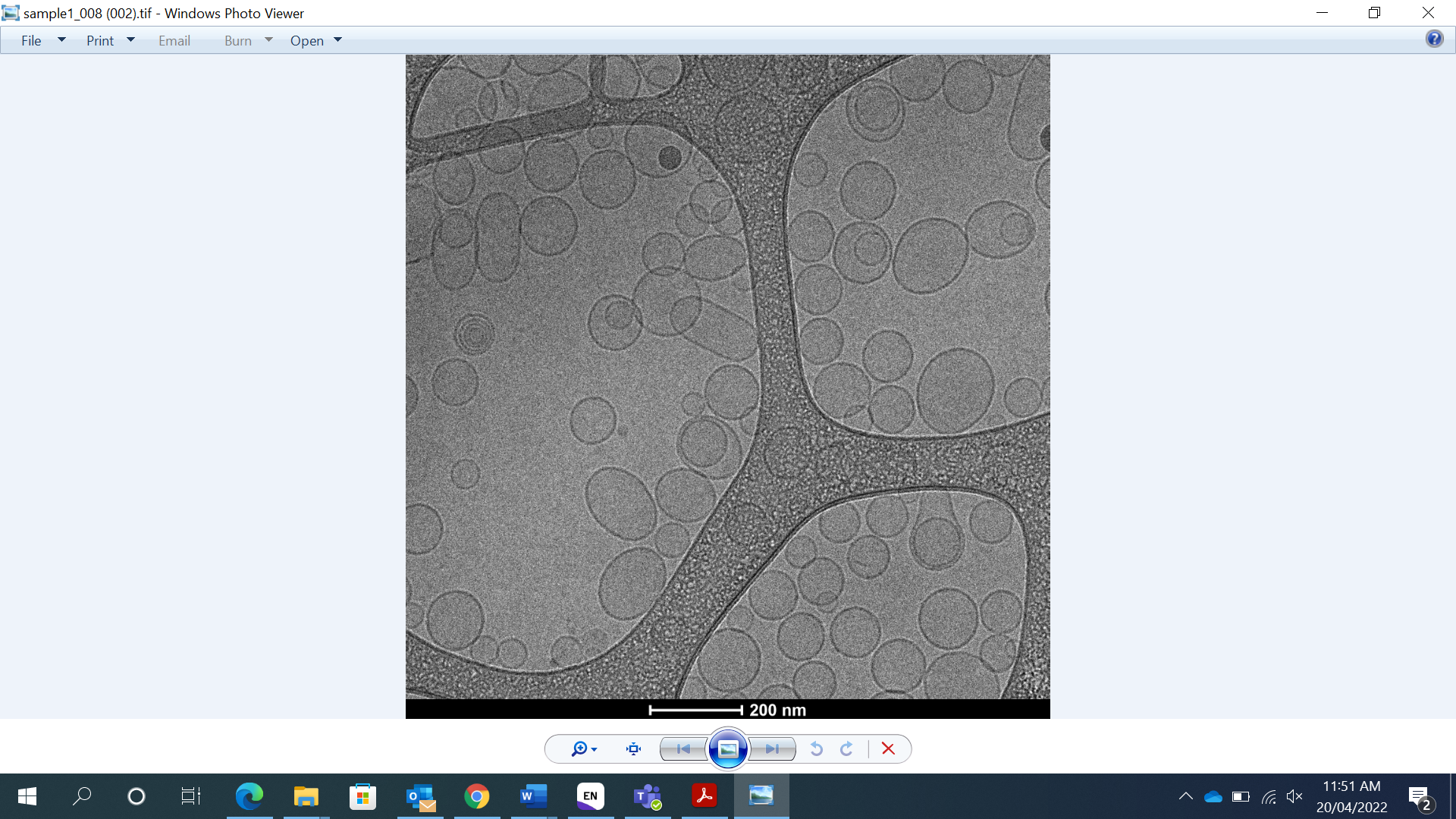


**Cryo-TEM image of non-radiolabeled liposomes.**

**Table of liposome characteristics before and after nebulization in a PARI vibrating mesh nebulizer for 20 mins. Data represents mean ± sd (n=3).**

|  | **Size (nm)** | **PDI** | **Zeta potential (mV)** |
| --- | --- | --- | --- |
| **Pre-nebulization** | 136 ± 1.1 | 0.14 ± 0.02 | 0.23 ± 0.17 |
| **Post-nebulization** | 166 ± 1.4 | 0.30 ± 0.01 | 0.68 ± 0.03 |

# Pre- and post-surgical infection control and pain relief

One day before surgery the inner right leg of sheep was cleaned, dried and a fentanyl patch (50/75 µg) applied to the skin to provide constant pain relief during and post-surgery. Intramuscular (IM) diazepam was administered to provide pre-surgical sedation followed by intravenous (IV) thiopentone (10 mg/kg) to induce anesthesia. Sheep were intubated with a cuffed endotracheal tube (Portex, 7-8mm), connected to a mechanical ventilator and maintained under anesthesia via isoflurane (1-5% in oxygen). Infection prevention was managed during surgery via the administration of IM procaine penicillin (400 mg) and IV cephazolin (1 g every 90 min). Subcutaneous (SC) injections of bupivacaine (5 ml) into the incision sites and into the 5^th^ intercostal space provided local anesthesia and nerve block.

**2 Quantification of ^3^H-lipid in plasma, organ, urine and feces samples**

In brief, samples of plasma, BALF, lymph fluid, urine and cell pellets from BALF and lymph fluid were mixed with 10 ml Ultima Gold^TM^ and analyzed via liquid scintillation counting. Feces were homogenized in water and dried to evenly disperse the radiolabel. An aliquot of dried feces (100 mg) was then digested in 2 ml of Soluene at 60°C overnight. Sodium hypochlorite (1 ml) was then added to bleach samples before adding Ultima Gold^TM^ (10 ml) and resting the samples in the dark at room temperature for at least 3 days before analysis via liquid scintillation counting. Organs were homogenized in water and 100 mg aliquots digested in 2 ml Soluene at 60°C overnight. Samples were then bleached in 200 ul 30% H_2_O_2_, mixed with Ultima Gold^TM^ and stored at room temperature for at least 3 days in the dark before being analyzed via liquid scintillation counting. All liquid scintillation was performed using a TriCarb 2000CA liquid scintillation counter (Packard TriCarb 2000CA, Meriden, CT). Blank organs and BALF (from undosed donor sheep), and urine and feces (collected pre-dose from each sheep) were similarly processed to perform background corrections.


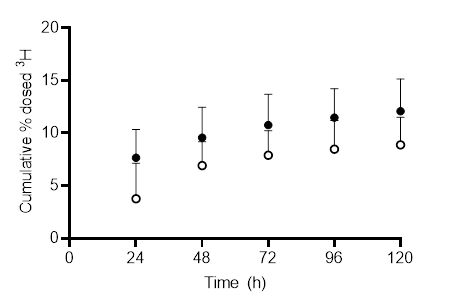

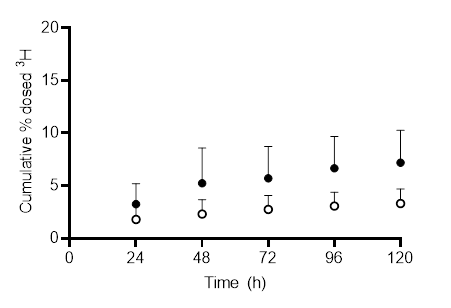


**B**

**A**

**Cumulative excretion profiles in feces (A) and urine (B) after IV (closed symbols) and pulmonary (open symbols) dosing of ^3^H-liposomes in sheep. Data represent mean ± sd (n=4­).**

**Organ:plasma ratios calculated as (ng lipid/g tissue) / (ng lipid/ml plasma) 5 days after pulmonary or IV dosing to sheep. Values represent mean ± sd (n = 4). *Represents p<0.05 cf. IV group via unpaired Student’s T-test.**

|  | **INTRAVENOUS** | **PULMONARY** |
| --- | --- | --- |
| **Lungs** | 3.4 ± 2.2 | 12.6 ± 2.2* |
| **Liver** | 3.0 ± 1.5 | 6.6 ± 0.3* |
| **Kidney** | 2.7 ± 0.8 | 3.3 ± 0.3 |
| **Spleen** | 3.3 ± 1.5 | 4.8 ± 1.6 |
| **CMLN** | 1.8 ± 0.6 | 2.8 ± 0.6 |
